# Virtual reality simulation for nursing education: effectiveness and feasibility

**DOI:** 10.1186/s12912-023-01639-5

**Published:** 2023-12-19

**Authors:** Debra Kiegaldie, Louise Shaw

**Affiliations:** 1 Eastern Health Clinical School, Monash University, Victoria, 3128 Australia; 2 Centre for Digital Transformation of Health, University of Melbourne, Grattan Street, Parkville, Vic 3010 Australia

**Keywords:** Virtual reality simulation, Simulation-based education, Scenario, Nursing education, Online

## Abstract

Virtual Reality simulation (VRS) is an innovative and emerging technology that has the potential to offer increased numbers of pre-registration students authentic learning experiences compared to traditional simulation- based education (SBE) with simulated participants. The aim was to evaluate learner outcomes of SBE compared to 4 fully immersive VRS scenarios, for vocational and higher education nursing students at a training and further education institute in Melbourne, Australia. A mixed methods quasi-experimental design study was conducted over two semesters from 2019 to 2020. Participants were 675 pre-registration nursing students. The intervention group (VRS n = 393) received 4 three-dimensional, immersive VRS modules. The control group (SBE n = 282) received 4 face-to-face large group immersive simulations. In the VRS group 95% of students actively participated, compared to SBE (on average 15%). Knowledge test scores were initially significantly greater (*p* < 0.01) for VRS versus SBE students, but not maintained post clinical placement. Intervention students found VRS to be realistic and prepared them for clinical practice. Some technical difficulties were identified with VRS. VRS was found to be more cost effective than SBE. VRS fostered critical thinking and provided an efficient and sustainable platform for learning about complex clinical situations.

**Supplementary Information:**

The online version contains supplementary material available at 10.1186/s12912-023-01639-5.

## Introduction

Most health professional pre-registration courses now make use of simulation-based education (SBE) as an evidence-based educational method to practise clinical skills [1–3]. Clinical simulation environments are immersive and multisensory, promoting the development of psychomotor skills and executive functioning [1]. Simulation became particularly valuable as an education method for meeting the challenges posed by the need for virtual learning during the COVID 19 pandemic [4]. Traditional simulations are highly successful but currently not scalable due to increasing demand, student numbers and access limitations [5]. Few students can actively participate in an immersive simulation, with the majority being inactive observers [5]. The ability to provide increased opportunities for all students to be involved in authentic work-based learning experiences is essential for students to become effective and competent practitioners.

Virtual Reality (VR) technology is an innovative and emerging technology that is increasingly being used in health professions education. VR simulations allow for active learning experiences that are interactive, authentic, standardised, and safe [6–8]. Three-dimensional VR simulations where there is a perception of being physically present in the virtual world [9, 10], are seen as being highly immersive [11]. Wearing VR glasses or head mounted displays, adds to the visceral feeling of being in the simulated world [10]. Once the VR scenarios have been created, they can be re-used multiple times for a variety of learners [6], and can be accessed by students anywhere at any time [7]. They allow students to experiment with different outcomes, practise the scenarios, and experience elevated risk events without compromising safety [7]. Limitations to VR include lack of immersion and realism [12]. VR scenarios can also be expensive to develop and time-intensive [6, 7, 13]. VR requires a fast and reliable internet service, computer skills, technology resources and appropriate training and orientation prior to commencement [7].

Whilst there is some evidence for the use of VR simulation (VRS) in student nursing education to increase students’ knowledge and positive perceptions of their learning experience [8, 14–16], there is limited literature on the economic viability of VRS. Jasper^VR^ was a fully immersive virtual reality education program, developed through a collaborative consortium, that immersed students’ senses (vision, hearing, and motion) in a 3D world for a range of common clinical scenarios. By using the VR headset and by means of gaze control, students were able to make decisions that in turn determined the future trajectory of each patient they interacted with.

The aim of this study was to evaluate learner outcomes of traditional SBE compared to a series of fully immersive VRS scenarios, for vocational and higher education nursing students at a training and further education institute in Melbourne, Australia.

Research questions:

1. What was the impact of VRS compared to SBE on students’ knowledge of, confidence in, and motivation to learn about managing common clinical conditions?
2. What are the reactions of students in relation to the usability, efficiency, and effectiveness of VRS?
3. What is the impact of VRS compared to SBE on the number of nursing students with an immersive simulation learning experience?
4. Is the use of Jasper^VR^ a feasible and economically viable education method?

## Materials and methods

Using a mixed methods quasi-experimental design, this study compared educational outcomes from traditional simulation using simulated participants with VRS. Ethical approval was obtained from Monash University Human Research Ethics Committee, Project ID: 19235. Written informed consent was obtained from the participants of the study.

### Materials

Jasper^VR^ was a uniquely developed VR-based education platform called VirtualU, that was developed through a collaborative consortium. Using 360-degree video and sound technology, the Jasper^VR^ VirtualU software application captured the variations and potential outcomes of a range of common clinical scenarios using simulated participants. By using the VR headset and by means of gaze control, students were able to select pre-determined actions, that in turn determined the future trajectory of each patient they interacted with. Feedback on patient outcomes following choices made by the student, was provided via visualisation of the different pathways. Debriefing and discussion took place after each simulation.

### Participants

A representative sample of consenting students enrolled in the Bachelor and Diploma of Nursing courses at a Training and Further Education Institute between July 2019 and June 2020, participated in the study. Consenting students consisted of 7 cohorts (Table 1). Students were pre-allocated to tutorial groups (n = 5–6), which were randomly assigned by an external organisation, to either the control or intervention groups. Students who did not consent to participate in the research remained in a non-intervention group and received standard teaching.

### Facilitators

For the duration of the research a core group of experienced facilitators (n = 3) were used for both the control and intervention groups. Facilitators received a 1-hour training session on how to debrief and were provided with a debriefing guide. Debriefing was based on the Promoting Excellence and Reflective Learning in Simulation (PEARLS) framework [17].

### Simulation scenarios (VRS and traditional simulation)

Module 1: The verbally aggressive patient, Module 2: The deteriorating patient, Module 3: The patient with cognitive impairment, Module 4: Palliative and end of life care.

### Interventions

#### Control group (traditional simulation)

students participated in standard teaching activities in the Diploma and Bachelor of Nursing, including lectures (large group), tutorials (small group), clinical skills laboratories, role-plays, and four face to face large group immersive simulation scenarios using simulated participants, in the Simulation Centre.

#### Intervention group (VRS)

students participated in the same standard teaching activities as the control group but received the four Jasper^VR^ modules as an alternative to traditional face to face large group immersive simulation scenarios.

#### Implementation

Healthcare simulation standards of best practice were utilised for both the intervention and control scenarios [18]. Whilst, pre-briefing and debriefing was delivered separately to the control and intervention groups, the intervention and control groups received the same pre-briefing and debriefing according to the PEARLS framework [17].

#### Pre-briefing for each module

An experienced and trained facilitator provided an overview and briefing to control or intervention students on the module’s content, key learning points and its application to clinical practice.

#### Debriefing

Control or intervention students received a scheduled debriefing session for each module from one of the experienced facilitators. The debrief provided an opportunity for students to explore the content in more depth, discuss important clinical issues, ask questions, and clarify important concepts.

#### Delivery of VRS

Intervention students were provided with a Jasper^VR^ handbook and VR headset. At the commencement of the first session students downloaded the required mobile software application, VirtualU, onto their mobile phone, using the education institute’s Wi-Fi. Students were provided technical assistance from the project managers for the duration of the research study.

Each VRS module had two options:

- *Free roam*: available within or external to classroom time, allowed students to navigate their way through the module scenarios, make decisions about clinical practice and reflect on critical issues that arose. They were able to repeatedly view the scenarios in preparation for clinical placement.
- *Mastery videos*: embedded into the program and included videos of clinical experts performing a range of skills to demonstrate exemplar performance of best practice. Students were able to identify key features of a good performance to help prepare them for clinical placement. In addition to offering students the opportunity to view the mastery videos in their own time, teachers could select to use this option in class to highlight aspects of high-quality performance.

#### Data collection

Multiple methods of data collection were used (Table 1).

**Table 1:** Protocol for between group comparisons on specific modules

Stage	Comparison Group	Intervention Group
Pre – Teaching	Student Survey 1 (Pre-test) • Student characteristics • Knowledge test • Knowledge scale • Motivation to learn scale • Self-efficacy confidence scale	Student Survey 1 (Pre-test) • Student characteristics • Knowledge test • Knowledge scale • Motivation to learn scale • Self-efficacy confidence scale
Semester	Traditional teaching including simulation	Traditional teaching plus Jasper^VR^ Software analytics
Post Teaching (Final Week of Semester)	Survey 2 (Post-test 1) • Knowledge test • Knowledge scale • Motivation to learn scale • Preparation for clinical placement scale • Self-efficacy confidence scale • Views and ratings on the simulation learning experience • Clinical assessment	Survey 2 (Post-test 1) • Knowledge test • Knowledge scale • Motivation to learn scale • Self-efficacy confidence scale • Preparation for clinical placement scale • Views and ratings on the Jasper^VR^ learning experience • System Usability Scale • Clinical assessment
Focus Group Interview		Focus group interview OR Individual telephone interviews
Post Clinical Placement	Survey 3 (Post-test 1) • Knowledge test • Knowledge scale • Motivation to learn scale • Preparation for clinical work scale • Self-efficacy confidence scale • Views on clinical placement experience	Survey 3 (Post-test 1) • Knowledge test • Knowledge scale • Motivation to learn scale • Preparation for clinical work scale • Self-efficacy confidence scale • Views on clinical placement experience
Economic Evaluation	End of Semester	

#### Surveys

The study was based on a pre and post-test design, elaborated through a mixed methods research approach. Data was collected via surveys at multiple time points.

Pre-test (Survey 1): Week 2–3 of semester prior to simulation week.

Post-test 1 (Survey 2): Final week of semester.

Post-test 2 (Survey 3): Post clinical placement.

Students completed surveys either online or paper based. Pre-test surveys included quantitative measures of knowledge (multiple choice questions), a self-reported knowledge scale (7-point Likert scale from ‘not at all knowledgeable’ to ‘extremely knowledgeable’), a motivation to learn scale (7-point Likert scale from ‘not at all motivated’ to ‘extremely motivated’), and a self-efficacy in learning scale. The self-efficacy in learning scale consisted of a range of 10–13 items with students indicating their level of confidence to perform skills for dealing with patients for each of the modules on a 5-point Likert scale from ‘not at all confident’ to ‘extremely confident’. Questions were based on the learning objectives for the Bachelor and Diploma of Nursing curriculum in relation to each topic. The items were designed collaboratively between researchers and the faculty teaching teams. They were checked for authenticity and content validity through peer review. Post-test surveys repeated these measures and added views and ratings on the learning experience (simulation or virtual reality). The items for the views on the learning experience were based on McCausland et al’s (2004) survey evaluating student experiences of simulation in undergraduate nursing [19] and used a 5-point Likert-scale from ‘strongly disagree’ to ‘strongly agree’. Post-test surveys for the VR intervention students also included the System Usability Scale (SUS). The SUS is a 10-item scale giving a global view of subjective assessments of usability using a 5-point Likert Scale [20]. The SUS has demonstrated robust psychometric properties and measured the overall usability of the VirtualU software application.

#### Focus group interviews

semi-structured interviews were conducted with intervention students that focused on the students’ views about their general learning experiences with Jasper^VR^, the lessons learnt and what could be improved.

#### Student clinical assessment

Students were asked to consent to using a de-identified course assessment of an Objective Structured Clinical Observation (OSCE), which they undertook at the conclusion of each semester, as a measure of the impact on clinical learning outcomes on actual performance related to specific clinical content of each module. These assessments were undertaken with simulated participants and assessments were directly aligned to the specific module content.

#### Economic evaluation

a cost benefit analysis was conducted to compare the cost and benefits of Jasper^VR^ with traditional SBE. The cost of immersive, mannequin and simulated patient-based simulation was calculated per student and a comparison made.

### Data analysis

#### Quantitative data

Using SPSS (IBM SPSS Statistics for Windows, Version 21.0), descriptive statistics identified demographic data. The chi-squared test was used to ensure that the demographics of the intervention and control groups were sufficiently equivalent. The remainder of the quantitative analysis used inferential statistics to test for systematic differences between outcomes for the control and intervention groups, and systematic differences within the intervention groups across the three time points (for 2019 groups). This was based on the application of several quantitative instruments. Statistical controls were performed using a two-way repeated measures ANOVA.

#### Qualitative data

Qualitative data from the open-ended questions in the surveys were transcribed verbatim. Quantitative data were analysed and reported thematically. Themes reflected intervention participants’ accounts of their learning experience with VRS and characterised perceptions that were relevant to the research questions [21, 22]. One researcher developed descriptive codes of the responses to each of the open-ended questions on the surveys. A different researcher reviewed the initial descriptive codes, and the two researchers discussed and finalised the thematic framework for analysis. Data management and analysis were assisted by MS Excel.

#### Economic evaluation

Costs were calculated according to the cost of developing and delivering traditional SBE and comparing this to the cost of developing and delivering Jasper^VR^ [23, 24].

## Results

The overall cohort for the comparison study comprised 675 students, from the aggregation of seven distinct teaching cohorts (Cohorts 1 to 7). Due to the COVID-19 global pandemic in 2020, the implementation of control groups and cross over groups was not able to be achieved and all students were allocated into intervention groups to receive the JasperVR learning experience. This was due to all simulations being cancelled in Semester 1, 2020. Furthermore, it was not possible to implement any Survey 3s and OSCEs in 2020 due to the overwhelming number of cancellations of clinical placements and clinical assessments for students. This resulted in 282 students in the traditional simulation control group and 393 in the VR intervention group. Table 2 describes the control and intervention groups resolved in terms of: Cohorts (1 to 7); course of study (BN or DN); Age Range (18–25 years and 26–50 + years); and Gender.

**Table 2:** Demographics of the overall cohort. The Pearson Chi-Square test indicated no statistically significant difference between the Control and Intervention groups based on: contribution from the different teaching cohorts (*c*^2^ = 10.4, *df* = 6, *p* = 0.108), course of study (*c*^2^ = 3.22, *df* = 1, *p* = 0.073), student status (*c*^2^ = 0.042, *df* = 1, *p* = 0.837), distribution of the age ranges (*c*^2^ = 0.043, *df* = 1, *p* = 0.836), or gender (*c*^2^ = 2.91, *df* = 1, *p* = 0.088)

Cohort	Control (Sim)	Interven. (VR)	Total N	Total %
Cohort 1	45	66	111	16.4
Cohort 2	29	31	60	8.9
Cohort 3	42	56	98	14.5
Cohort 4	23	54	77	11.4
Cohort 5	91	99	190	28.1
Cohort 6	34	63	97	14.4
Cohort 7	18	24	42	6.2
Total	282	393	675	100.0
**BN or DN**	**Control (Sim)**	**Interven. (VR)**	**Total** **N**	**Total** **%**
BN	178	221	399	59.1
DN	104	172	276	40.9
Total	282	393	675	100.0
**Age Range**	**Control (Sim)**	**Interven. (VR)**	**Total** **N**	**Total** **%**
18–25 yr	180	263	443	65.6
26–50 + yr	90	127	217	32.1
Missing	12	3	15	2.2
Total	282	393	675	100.0
**Gender**	**Control (Sim)**	**Intervention (VR)**	**Total** **N**	**Total** **%**
Male	62	70	132	19.6
Female	203	320	523	77.5
Other	2	0	2	0.3
Missing	15	3	18	2.7
Total	282	393	675	100.0

### Simulation participation

Approximately 95% of students actively participated in the VR scenarios. Approximately 3–5% of students were not able to participate due to incompatible phones and therefore were transferred to the traditional simulation control group. Approximately 15% of students in the traditional simulation control group actively participated in the simulation scenarios with 85% observers, which is standard practice at the participating institute.

### Knowledge test results

Pre-intervention there was nothing to suggest any systematic difference between the knowledge test results of the control group and of the intervention group. For the post-test (survey 2), 15 out of 17 *t*-tests returned positive results at the level of *p* < 0.001, suggesting that the intervention group performed better than the control group in the knowledge test (Table 3).

In survey 3, there was no significant difference between the knowledge test results of the control group and of the intervention group. The results indicate that any difference in knowledge was not maintained post clinical placement.

**Table 3:** Knowledge Test, Questions Q1 – Q10 responses pooled to give Total Score/10. Descriptive statistics (columns 4–7; N, Mean, StdDev and 95% Confidence Interval) for Control and Intervention Groups

Selection Property		Group	Descriptive Statistics				t-test for Equality of Means		
			N	Mean	Std. Dev.	95% C.I.	t	df	Sig.
**All**	All Cases	Control (Sim)	734	5.75	2.15	0.159	-1.4	1598.4	0.169
		Intervention (VR)	1115	5.89	2.21	0.132			
**Module**	Module 1	Control (Sim)	157	7.85	1.49	0.238	-1.8	279.8	0.080
		Intervention (VR)	267	8.10	1.23	0.150			
	Module 2	Control (Sim)	232	5.21	1.48	0.194	-0.9	527.7	0.361
		Intervention (VR)	327	5.33	1.65	0.182			
	Module 3	Control (Sim)	245	4.36	1.87	0.240	0.9	484.9	0.382
		Intervention (VR)	364	4.23	1.68	0.176			
	Module 4	Control (Sim)	100	7.09	1.62	0.324	-0.2	219.3	0.813
		Intervention (VR)	157	7.14	1.71	0.273			
**Survey 2, Section B, Q1-10, total score/ 10 (cohorts 1–6)**									
**All**	All Cases	Control (Sim)	511	6.09	2.24	0.20	-9.1	928.7	< 0.001**
		Intervention (VR)	810	7.16	1.84	0.13			
**Module**	Module 1	Control (Sim)	129	7.73	1.60	0.28	-4.3	194.8	< 0.001**
		Intervention (VR)	223	8.41	1.07	0.14			
	Module 2	Control (Sim)	129	5.68	1.78	0.31	-6.7	235.8	< 0.001**
		Intervention (VR)	217	6.93	1.51	0.21			
	Module 3	Control (Sim)	183	4.92	2.14	0.32	-3.9	346.2	< 0.001**
		Intervention (VR)	239	5.69	1.75	0.23			
	Module 4	Control (Sim)	70	6.84	2.21	0.53	-4.4	99.3	< 0.001**
		Intervention (VR)	131	8.13	1.40	0.24			
**Survey 3, Section B, Q1-Q10 (cohorts 3, 4 and 6)**									
**All**	All Cases	Control (Sim)	270	5.88	2.68	0.33	-0.6	570.9	0.541
		Intervention (VR)	442	6.00	2.69	0.26			
	Module 1	Control (Sim)	75	8.24	1.00	0.23	-1.3	155.7	0.188
**Module**		Intervention (VR)	120	8.43	0.99	0.18			
	Module 2	Control (Sim)	72	5.94	1.91	0.45	-1.1	136.0	0.257
		Intervention (VR)	121	6.26	1.71	0.31			
	Module 3	Control (Sim)	75	2.55	1.31	0.30	0.4	135.8	0.710
		Intervention (VR)	121	2.48	1.09	0.20			
	Module 4	Control (Sim)	48	7.29	1.61	0.46	-0.1	104.2	0.945
		Intervention (VR)	80	7.31	1.72	0.38			

### Students’ self-perceived knowledge and motivation

The results show immediately post intervention, the intervention group’s ratings for self-perceived knowledge, motivation and preparedness for clinical placement were more positive than the control group at the *p* < 0.01 level (Table 4). The differences between the control and interventions groups were not maintained post clinical placement.

**Table 4:** Students’ self-perceived knowledge, motivation and preparedness for clinical placement or clinical practice. Descriptive statistics and Independent Samples t-test (All Cohorts. All Modules pooled. Surveys 2 and 3)

Survey 2 Question	Group	Descriptive Statistics				t-test for Equality of Means		
		N	Mean	Std. Dev.	95% C.I.	t	df	Sig.
KNOWLEDGE	Control (Sim)	493	4.57	1.495	0.134	-9.99	903.3	< 0.001**
	Intervention (VR)	786	5.37	1.240	0.088			
MOTIVATION	Control (Sim)	493	5.73	1.134	0.102	-6.04	968.0	< 0.001**
	Intervention (VR)	786	6.11	1.027	0.074			
PREPAREDNESS (for clinical placement)	Control (Sim)	493	5.03	1.163	0.104	-6.57	1018.1	< 0.001**
	Intervention (VR)	786	5.46	1.124	0.080			
**Survey 3** **Question**	**Group**	**Descriptive Statistics**				**t-test for Equality of Means**		
		**N**	**Mean**	**Std. Dev.**	**95% C.I.**	**t**	**df**	**Sig.**
KNOWLEDGE	Control (Sim)	268	5.10	1.097	0.134	-0.45	515.35	0.653
	Intervention (VR)	439	5.14	0.978	0.094			
MOTIVATION	Control (Sim)	268	6.00	1.067	0.130	0.532	543.143	0.595
	Intervention (VR)	440	5.96	1.017	0.096			
PREPAREDNESS (for clinical practice)	Control (Sim)	245	5.28	1.161	0.148	0.48	464.156	0.632
	Intervention (VR)	401	5.23	1.017	0.102			

### Self-efficacy in learning

Immediately post intervention, the intervention group’s self-efficacy in learning was more positive than the control group’s for modules 3 and 4 and the *p* < 0.01 level. However, differences between the intervention and control groups were not maintained post clinical placement (Table 5).

**Table 5:** Self-efficacy in learning, pooled responses to give Total Score/100, results resolved according to module number (1–4). Descriptive statistics (columns 4–7; N, Mean, StdDev and 95% Confidence Interval) for All Cohorts (i.e., both BN Cohorts and DN Cohorts), for Control and Intervention Groups. Independent Samples t-test comparing the mean values for the Control and Intervention groups. ** indicates significance at the *p* < 0.01 level

Selection Property		Group	Descriptive Statistics				t-test for Equality of Means		
			N	Mean	Std. Dev.	95% C.I.	t	df	Sig.
**Survey 2**	Module 1	Control (Sim)	121	81.00	10.05	1.83	-1.3	260.2	0.183
		Intervention (VR)	217	82.56	10.63	1.44			
	Module 2	Control (Sim)	130	78.78	10.57	1.85	-0.6	309.3	0.545
		Intervention (VR)	217	79.55	12.65	1.72			
	Module 3	Control (Sim)	182	70.04	14.96	2.22	-6.4	352.3	< 0.001**
		Intervention (VR)	238	78.91	12.63	1.64			
	Module 4	Control (Sim)	71	76.08	14.00	3.32	-4.1	117.1	< 0.001**
		Intervention (VR)	131	83.97	10.94	1.91			
**Survey 3**	Module 1	Control (Sim)	75	81.29	9.77	2.26	-0.2	166.3	0.821
		Intervention (VR)	120	81.63	10.55	1.93			
	Module 2	Control (Sim)	72	76.00	12.27	2.89	0.3	160.6	0.787
		Intervention (VR)	121	75.48	13.48	2.45			
	Module 3	Control (Sim)	73	72.05	14.89	3.49	-1.1	137.3	0.256
		Intervention (VR)	121	74.46	13.14	2.39			
	Module 4	Control (Sim)	48	75.00	12.09	3.49	-1.1	100.3	0.289
		Intervention (VR)	78	77.37	12.20	2.76			

### Analysis of OSCE data

OSCE data was obtained for 478 students (control n = 177, intervention n = 301). The Pearson Chi-Square test (c^2^ = 0.267, df = 2, *p* = 0.875) indicated there was no statistically significant difference between the control and intervention groups.

For Modules 1 and 2 there was no significant difference between the Mean OSCE score (as a %) for the control and intervention groups. However, for Module 3 the Mean OSCE score for the intervention group was significantly greater than that for the control group, at the *p* < 0.01 level. When all three Modules were pooled together, while the OSCE score was greater for the intervention group than for the control group, the difference was not significant at the *p* < 0.05 level (Table 6).

**Table 6:** Mean OSCE Score (as %, StdDev and 95% Confidence Interval) for all cases (n = 478), for Control and Intervention Groups

Module Number	Group	Descriptive Statistics				t-test for Equality of Means		
		N	Mean Score %	Std. Dev.	95% C.I.	t	df	Sig.
**Module 1**	Control (Sim)	30	79.8	16.6	6.06	0.726	43.3	0.472
	Intervention (VR)	51	77.3	10.7	2.98			
**Module 2**	Control (Sim)	99	70.0	11.1	2.23	− 0.790	241.9	0.431
	Intervention (VR)	162	71.3	14.0	2.19			
**Module 3**	Control (Sim)	48	49.1	17.7	5.10	-3.606	94.3	0.000**
	Intervention (VR)	88	60.4	17.2	3.66			
**Pooled Modules**	Control (Sim)	177	66.0	17.8	2.67	-1.931	332.3	0.054
	Intervention (VR)	301	69.1	15.7	1.80			

### Views about the module (survey 2)

All *t*-tests comparing mean values for the control and intervention groups for views on the modules returned positive results at the level of *p* < 0.01, with three of them at the level of *p* < 0.001 (Table 7).

**Table 7:** Views about the module, responses pooled to give Total Score/50, (cohorts 1–6 -no data cohort 7)

Selection Property		Group	Descriptive Statistics				t-test for Equality of Means		
			N	Mean	Std. Dev.	95% C.I.	t	df	Sig.
**All**	All Cases	Control (Sim)	382	40.37	6.59	0.67	-8.9	665.4	< 0.001**
		Intervention (VR)	791	43.88	5.71	0.41			
**Module**	Module 1	Control (Sim)	77	41.34	6.41	1.46	-2.8	125.6	0.005**
		Intervention (VR)	218	43.72	5.98	0.81			
	Module 2	Control (Sim)	125	41.32	5.22	0.93	-3.9	272.0	< 0.001**
		Intervention (VR)	205	43.67	5.48	0.77			
	Module 3	Control (Sim)	127	39.65	6.89	1.22	-5.1	222.8	< 0.001**
		Intervention (VR)	237	43.35	5.80	0.75			
	Module 4	Control (Sim)	53	38.42	8.32	2.28	-5.7	69.1	< 0.001**
		Intervention (VR)	131	45.44	5.21	0.91			

### Qualitative analysis

Several common themes were identified across the 4 modules for intervention students’ responses on views about the learning experience with Jasper^VR^ (Table 8). Students reported that they found the VRS scenarios realistic. They valued the ability to be able to practise the various scenarios multiple times to embed their learning and highlighted the importance of being able to make mistakes without fear of impacting care. The VRS scenarios were perceived to be less stressful and intimidating than the usual simulation environment. The value of developing critical thinking from the VRS scenarios was highlighted and the development of the necessary knowledge and skills to be able to manage similar situations.

While student responses were mostly positive and enthusiastic about the VR scenarios, they did express some reservations. Occasionally the VR headsets were reported to be uncomfortable to wear. Whilst some students found the VRS scenarios less intimidating than a normal simulation, a small number of students described their desire to be more physically involved, rather than watching and choosing options. A small number of students also reflected that their clinical reasoning skills would have benefitted from there being more than one correct choice of response, learning diverse ways to manage situations, or having multiple scenarios for each module. Further detail on students’ views on what they enjoyed the most and least for each module are detailed in appendix 1.

**Table 8:** Qualitative data for intervention students’ views on Jasper ^VR^.

Module	Theme	*Quote*
**Enjoyed the most**		
**Common feedback (all modules)**	Realism	*Felt like a real situation and it helped me to handle any situation in the future.* *The scenario was realistic, and the module was able to give you a chance to reflect on the options you have chosen to manage the situation.* *Use of different locations and scenarios made the virtual experience more realistic*
	Rehearsal, ability to make mistakes	*The information that was delivered was relevant. I loved the systematic delivery of information. I could choose wrong answers and know how the patient would react.* *… I liked that it told you what you did right and what you didn’t. You get to see the different things that can happen if you don’t choose the right choice which is educational.* *The existence of ‘mastery mode’ -the best solution for the situation and how a professional would deal with the situation.* *Choosing the wrong choices leads you to well performed scenes. Actors took the time to show all paths*
	Reduced stress and increased confidence compared to a normal SIM	*I was not there physically to witness the scene. It was less intimidating.* *The VR was fantastic compared to the usual physical sim. The VR environment was student-friendly, did not cause any anxiety and therefore, helped learn a lot.* *I was able to maintain calm and well settled behaviour due to confidence build up through our Jasper activities*
	Ability to practise the modules multiple times	*Not being stressed out having the opportunity to practise as many times as I want.*
	Development of critical thinking	*The fact that I felt well immersed into the environment of the simulation. A proper handover was delivered and gave me direction of how I could manage the patient.* *The VR project gave … me options to choose from which promoted higher order critical thinking and reasoning skills to be used in practice within the simulated scenario*
**Enjoyed the least**		
**Common feedback (all modules)**	Technical: VR headset	*The VR headsets did not work well, was better able to watch using mobile view mode.* *Using the VR-GX device. Using normal view on my phone was more enjoyable. It was difficult with wearing glasses*
	Scenario: Lack of interaction	*The thing I least enjoyed was not being able to interact with the patient and the situation.* *Not having the chance to do skills by my own hand. Having to stand on a corner and watch the whole situation.* *I know it’s hard, but I wanted to be more involved* *Break up sections with more interactive parts to better take in all the information*
	Scenario: Development of clinical reasoning	*Wish there were more questions and options to choose and participate.* *More options that could potentially be helpful, not just one right way*
	Scenario: Revision of information presented	*Add multiple choice questions after the scenario to recap the covered information presented in the scenario.*
	Additional scenarios required in different settings	*Maybe in a hospital setting with triggers that will cause patient to escalate (e.g., postponed surgery, etc.)* *Provide multiple video situations because everyone’s anger/aggression is different. Provide video of angry patient in room, as that situation is what we will most likely experience.* *I still want to learn and want content of deteriorating patient condition. Need more depth about this topic in regard to VR simulation.*

### System usability scale

The mean scores of the pooled usability scale are very complimentary of the usability of the system, particularly its ‘ease of use’ (Q3) and its property of being ‘easy to learn quickly’ (Q7) (Table 9).

**Table 9:** System Usability Scale responses. Descriptive statistics (N, mean value, standard deviation and 95% confidence interval) for the pooled cohort (N = 306, Cohorts 1,2,3,4,5 and 6 combined) responses

Question #	Question Text	Cohort	N	Mean	Std. Dev.	95% C.I.
Q1	I think that I would like to use this system frequently	All	305	4.06	0.97	0.11
Q2	I found the system unnecessarily complex	All	306	2.11	1.19	0.14
Q3	I thought the system was easy to use	All	306	4.22	0.92	0.10
Q4	I think that I would need the support of a technical person to be able to use this system	All	306	2.12	1.25	0.14
Q5	I found the various functions in this system were well integrated	All	305	3.95	0.93	0.11
Q6	I thought there was too much inconsistency in this system	All	306	2.02	1.06	0.12
Q7	I would imagine that most people would learn to use this system very quickly	All	305	4.19	0.87	0.10
Q8	I found the system very cumbersome to use	All	300	2.53	1.25	0.14
Q9	I felt very confident using the system	All	306	4.29	0.82	0.09
Q10	I needed to learn a lot of things before I could get going with this system	All	306	2.27	1.28	0.15

These findings were supported by the qualitative data in the surveys. Intervention students appreciated the ability to re-visit the VR scenarios as often as they liked and in their own time:

*I really enjoyed being part of Jasper*^*VR*^*and I felt it was a really nice way to learn the situation… best thing was I can rewatch it as many times as I want.*

*This was a great way to complete a simulation under the circumstances and a great way to continue this in the future as we have the luxury of completing it as many times as we will.*

Intervention students also found the VRS scenarios less intimidating than traditional simulation and liked the ability to be able to make mistakes and see the consequences of those choices:

*I really enjoy doing VR. I find it interesting and less stressful than doing a simulation with your whole class watching you and in front of actors/actresses. I also like how you can see what would happen if you chose the wrong choice in the situation and that you can do the VR as much as you want.*

Occasionally students reported difficulties with the VR headsets:

*I like the way that we could revisit the SIM whenever we wanted to, but I found controlling the SIM with the headwear hard to manage.*

### Feasibility and economic viability of Jasper^VR^ compared to SBE

Table 10 depicts the cost of developing and delivering the VR and SBE for both the Bachelor and Diploma of Nursing groups. The total cost of delivering a single module of Jasper^VR^ each year was $3,350. The cost of delivering a single SBE scenario (not necessarily the same) in nursing for both courses each year was $18,670.

**Table 10:** The cost of delivering the VR and SBE for the Bachelor and Diploma of Nursing groups

	Jasper^VR^ (costs per individual scenario)			Bachelor of Nursing			Diploma of Nursing		
One-off work	Item	Hrs	Cost	Item	Hrs	Cost	Item	Hrs	Cost
	Script development and review	160	$8,000	Script development	20	$1,000	Script development	20	$1,000
	Rehearsals	40	$2,000	Document review	12	$600	Document review	8	$400
	Filming (1 day) crew		$3,000						
	Filming (1 day) actors	64	$2,560						
	Filming (1 day) teachers	10	$500						
	Document update	10	$500						
	Software development	160	$8,000						
	Testing and QA	160	$8,000						
	Project development overhead	60	$3,000						
**Totals**		**664**	**$35,560**		**32**	**$1600**		**28**	**$1400**
**On-going use**	**Item**	**Hrs**	**Cost**	**Item**	**Hrs**	**Cost**	**Item**	**Hrs**	**Cost**
	Software license		$2,000	Preparations	23	$1,150	Preparations	16	$800
	Admin preparation	15	$750	Sim day – teachers	48	$2,400	Sim day – teachers	32	$1,600
	Pre-brief students	4	$200	Sim day – actors	21	$840	Sim day – actors	32	$1,280
	Debrief students	4	$200	Sim day – others (sim techs)	10	$500	Sim day – others (sim techs)	10	$500
	Technical support	4	$200	Additional remedial sim day (depending on group size	102	$5,100	Additional remedial sim day (depending on group size	190	$4,500
**Totals**			**$3,350**		**204**	**$9,990**		**180**	**$8,680**

## Discussion

Our study demonstrated that VRS provided authentic and positive learning experiences for nursing students. Participants indicated that they found the VR scenarios realistic, immersive, and aided in the development of their clinical reasoning. Authenticity in VR scenarios is important for preparation for the reality of clinical practice [25]. Our outcomes also support the view that VRS can be successfully employed to teach explicit behavioural skills such as teamwork, and decision-making [25, 26]. The VR scenarios catered for greater student numbers when compared to traditional simulation. All VRS students were fully immersed in the scenarios and able to take an active learning role the decision-making process. Jasper^VR^ was found to be a sustainable and cost-effective alternative to SBE.

Overall, this study found VRS to effective for improving student knowledge and performance, which is supported by other studies on the effects of VRS in nursing education [8, 14–16, 27]. Several factors can increase the cognitive load in VR scenarios compared to SBE. VR learners may initially need to process a large amount of sensory information due to a highly immersive experience, engagement of multiple senses simultaneously (for example, vision and hearing), mastery of controllers to interact with the virtual environment, and understanding and navigating 3D space. Our findings also reinforce the effectiveness of immersive VRS for improved cognitive load in nursing education, and that VRS is an effective teaching tool [9, 26].

VRS students’ OSCE scores for module 3, the patient with cognitive impairment, were significantly greater than for traditional simulation at the end of semester, although this difference was not maintained post clinical placement. For this scenario, students were particularly complimentary about the communication and collaborative skills amongst the interprofessional team and being able to practise skills such as recording the handover and assessing the patient. Active learning methods have previously been found to facilitate the development of logical reasoning [28] and reflective thinking [29]. The opportunity to perform similar skills in future VR scenarios may contribute to their further success. The significant differences in knowledge test scores between the VRS and traditional simulation groups immediately post intervention were not maintained. VR scenarios have the advantage of being available for users to access at any time point with easy repetition of scenarios, [30], and therefore VR students in may need to be reminded to re-visit these to further consolidate their learning and achieve proficiency.

Students appreciated the opportunity the VR scenarios provided to develop clinical competencies in authentic clinical experiences and see new perspectives, whilst maintaining their own safety and that of their patients [9]. VR simulations can offer a high level of realism and immersion compared to traditional SBE, with the learning process accelerated as students can experience scenarios that closely mimic real-life situations. The use of VRS in nursing education has been found to influence positive learning outcomes, such as stimulating interactivity and motivation amongst participants [25, 31, 32]. Sim et al. (2022) found that VRS enhances delivery of content related to patient care management (Sim et al., 2022). VR students valued access to the mastery mode to learn strategies like de-escalation techniques for dealing with an aggressive patient. Enabling VR students to practise skills through repetition, may allow them to master the skills more efficiently and achieve proficiency, versus SBE which has limitations in terms of repetition. VRS has been found to be particularly useful in situations such as dealing with aggressive patients as it protects the health professional’s safety and allows them to take a ‘trial and error’ approach to learning how to respond in these situations [33].

Whilst a small number of students highlighted a lack of interaction in the scenarios and being unable to practise the clinical skills in person, others reported the value of a fully immersive environment, such as being able to assess the deteriorating patient and recording the handover. Simulating the management of an acutely deteriorating patient has previously been successfully implemented in a nursing curriculum, with participants finding the simulation to be realistic and prepared them for clinical practice [34]. VR students also appreciated the opportunity for easy repetition of scenarios, experiencing the consequences of making wrong decisions, and the value of this for their learning. Through the VRS, nursing students could develop greater self-awareness and modify their reactions to a situation [33]. The importance of VRS for learning non-clinical skills by being able to ‘get it wrong to get it right’ was also highlighted in a study of undergraduate nursing students [25]. VR students found participating in the scenarios less intimidating than a normal simulation and appreciated the psychological safety involved in the learning [32]. This feedback echoed the findings of other VR studies that reported students using VR were less anxious than control groups when performing in the real world [35, 36]. Students also appreciated the ability to make mistakes in the virtual environment without fear of consequences [32].

Whilst the VR scenarios provided immediate feedback to the students and allowed them to correct their mistakes in real time, some students commented that there was only one correct option in the scenarios. They felt it would be more beneficial if the scenarios allowed a greater range of choices for patient management to more reflect the real world. Another study involving a VR scenario depicting unnecessary patient demand for antibiotics from a general practitioner, also found some scepticism amongst participants that VR technology could reflect the diversity and complexity of patient responses [33]. A system of learning for health professionals that involves menu-based actions, may mean that the student does not develop critical clinical reasoning skills [37]. VR simulations can be customised to meet the needs of learners and to meet specific learning objectives, which should be considered when developing further VR scenarios.

Participants in this study were generally positive about the usability of the VR technology, which echoes the findings of other immersive VRS nursing studies [11, 38]. Usability in terms of ease of use and users’ level of satisfaction are important characteristics of learning using virtual reality [7].

When considering the economic viability of VRS, the cost effectiveness of VR versus SBE can vary depending on several factors, including initial investment, maintenance, scalability, and accessibility. A previous study found the cost-utility ratio of virtual simulation (US$1.08) to be lower compared to mannequin-based simulation (US$3.62) [39]. In this study, the VR required significant upfront costs for software, hardware and development and was therefore more costly to develop when compared to standard SBE. This concurs with the findings of Liaw et al. (2018) who found that funding was important for the development and evaluation of virtual worlds in nursing education, due to the high outlay costs during design and development phases [40]. The VR simulations may also require ongoing software updates and maintenance costs, whilst the SBE may require periodic equipment replacement. However, our economic evaluation found the long-term delivery costs of VR to be significantly reduced due to less on campus teaching time and more independent learning for students. As reported by Pottle (2019) the costs of simulation are difficult to define, vary widely between institutions and are frequently under-reported [41]. Therefore, further studies are required which evaluate the cost effectiveness of VR compared to SBE for nursing students [30]. Our study found VR simulations required more time initially for learners to become comfortable with the technology and to set up the VR equipment, when compared to SBE. However, the virtual simulations are more scalable, allowing a larger number of students to be served simultaneously and to be used in further studies. VR simulations can be accessed remotely, offering flexibility for students, and making them more convenient for students who may not have easy access to physical simulation labs. This can save time and eliminate the need for travel, compared to SBE which usually requires students to be physically present in a specific location. As a result of COVID-19 in 2020, all learning with Jasper^VR^ moved to remote delivery. This resulted in an even further reduction in the hours associated with briefing and debriefing.

### Limitations

This study focused on undergraduate students and therefore further research is required to explore its application across the healthcare professional continuum. Some students felt their clinical judgement would benefit from having additional options rather than only one correct way of doing things. In future research, students could be given the opportunity to be included in the development of scenarios to gain their perspectives. There should be repeated exposure to similar situations to gain confidence in appropriately responding in real life. Future work could focus on students’ comments related to limited opportunities for hands on work in the VR scenarios, reduced patient interactions, and how these findings translate in the clinical setting. Due to the COVID-19 global pandemic in 2020, the implementation of control groups and cross over groups was not able to be achieved and all students were allocated into intervention groups to receive the Jasper^VR^ learning experience. This was due to all simulations being cancelled in Semester 1, 2020. Furthermore, it was not possible to implement any Survey 3s and clinical assessments in 2020 due to the overwhelming number of cancellations of clinical placements and clinical assessments for students. This resulted in many more students in the intervention group than the control group.

## Conclusions

Through a collaborative content and software development process, a sophisticated, scalable, highly usable, and authentic learning experience was created for pre-licensure nursing students. Jasper^VR^ enabled increased numbers of students to actively participate in an immersive simulated learning environment at their own pace, in their own time and venue. Jasper^VR^ fostered critical thinking and decision making and provided an efficient, cost effective and sustainable platform of learning for future nursing students. The VR simulations offered advantages in terms of immersive, repeatable, and feedback-rich experiences. Jasper^VR^ provided an efficient, cost effective and sustainable platform for learning for future nursing students.

### Electronic supplementary material

Below is the link to the electronic supplementary material.

**Appendix A**: qualitative data of intervention students’ responses to open ended questions on the surveys

## Data Availability

The data that support the findings of this study will be available from the corresponding author, LS, upon reasonable request.
