# Supplementary material for: Virtual reality simulation for nursing education: effectiveness and feasibility
Source: BMC Nurs. 2023 Dec 19;22:488. doi: 10.1186/s12912-023-01639-5 (PMC10729454; doi:10.1186/s12912-023-01639-5)
Supplement: Supplementary file 1 — Appendix A: qualitative data of intervention students’ responses to open ended questions on the surveys [file 12912_2023_1639_MOESM1_ESM.docx]

| **Module** | **Theme** | ***Quote*** |
| --- | --- | --- |
| **Enjoyed the most** | | |
| **Common feedback (all modules)** | Realism | *Felt like real situation and it helped me to handle any situation in the future.*  *The scenario was realistic and the module was able to give you a chance to reflect on the options you have chosen to manage the situation*  *Use of different locations and scenarios made the virtual experience more realistic* |
|  | Rehearsal, ability to make mistakes | *The information that was delivered was relevant. I loved the systematic delivery of information. I could choose wrong answers and know how the patient would react*  *… I liked that it told you what you did right and what you didn't. You get to see the different things that can happen if you don't choose the right choice which is educational*  *The existence of 'mastery mode' -the best solution for the situation and how a professional would deal with the situation*  *Choosing the wrong choices leads you to well performed scenes. Actors took the time to show all paths* |
|  | Reduced stress and increased confidence compared to a normal SIM | *I was not there physically to witness the scene. It was less intimidating*  *The VR was fantastic compared to the usual physical sim. The VR environment was student-friendly, did not cause any anxiety and therefore, helped learn a lot*  *I was able to maintain calm and well settled behaviour due to confidence build up through our Jasper activities* |
|  | Ability to practise the modules multiple times | *Not being stressed out having the opportunity to practise as many times as I want.* |
|  | Development of critical thinking | *The fact that I felt well immersed into the environment of the simulation. A proper handover was delivered and gave me direction of how I could manage the patient*  *The VR project gave … me options to choose from which promoted higher order critical thinking and reasoning skills to be used in practice within the simulated scenario* |
| **Module 1** | Learning de-escalation strategies | *It was a real experience. It was good to see how your speech and tone and words can help in calming someone.*  *How to deal with verbally aggressive person, use techniques to de-escalate them, calm them down, your safety. Scenario looked real.* |
|  | Use of mastery mode | *The mastery module where a professional individual de-escalated the patient was helpful to get a clear idea on a positive scenario.*  *The mastery mode that allowed me to understand how someone else would diffuse the situation* |
| **Module 2** | Teamwork | *I really enjoyed watching how a MET call scenario unfolded and the individual involvement of each team member.*  *I could actually see what is happening in a MET call situation. How leader nurse delegated roles to nurses and their managements.* |
|  | MET call process | *The multi layered approach to a MET call was helpful to experience. Having the family present was another element that was new to experience*  *The 'ABCDE' emergency assessment tool being used, and the doctor verbalising it louder to the team.* |
| **Module 3** | Learning strategies for how to manage a patient with delirium | *Scenario was well presented. Coordination and communication skills were good among nurses. Teach us how to handle cognitively impaired patients*  *Looking at how well the communication flowed in the room. Everyone worked together. Also having different ways in dealing with the scenario*  *The scenario was realistic. I learned new knowledge like we should not provide anti-psychotic drugs to delirious patients due to their side effects and the importance on including family members in the care of the patient* |
|  | Ability to practise required skills | *Being able to assess the patient, recording the handover and history and differentiate the situation*  *Learning how to deal with the patient in that situation* |
| **Module 4** | Seeing the full process of palliation | *Setting up the family meeting, giving choices for the patient for the pain management. The senior doctor demonstrated very well how to disclose the bad news to the client and family members*  *The nurse listening to patient and arranging a session with the senior doctor and the patient’s daughter to discuss test results.*  *Module 4 showed different situations and different timeline in the life of the patient, which is not seen it performed as an actual simulation or an actual situation. It gave us an insight of what should be done to this patient family during his different stages of the illness until death.* |
| **Enjoyed the least** | | |
| **Common feedback (all modules)** | Technical: VR headset | *The VR headsets did not work well, was better able to watch using mobile view mode*  *Using the VR-GX device. Using normal view on my phone was more enjoyable. It was difficult with wearing glasses* |
|  | Scenario: Lack of interaction | *The thing I least enjoyed was not being able to interact with the patient and the situation*  *Not having the chance to do skills by my own hand. Having to stand on a corner and watch the whole situation*  *I know it’s hard but I wanted to be more involved*  *Break up sections with more interactive parts to better take in all the information* |
|  | Scenario: Development of clinical reasoning | *Wish there were more questions and options to choose and participate*  *More options that could potentially be helpful, not just one right way* |
|  | Scenario: Revision of information presented | *Add multiple choice questions after the scenario to recap the covered information presented in the scenario* |
|  | Additional scenarios required in different settings | *Maybe in a hospital setting with triggers that will cause patient to escalate (e.g., postponed surgery, etc)*  *Provide multiple video situations because everyone's anger/aggression is different. Provide video of angry patient in room, as that situation is what we will most likely experience*  *I still want to learn and want content of deteriorating patient condition. Need more depth about this topic in regards to VR simulation* |
| **Module 1** | Receptionist’s attitude towards the patient | *Receptionist getting aggressive with the patient and raising her voice. It makes client more agitated.* |
| **Module 3** | Scenario: distance from the action felt too far away | *I don't like being in the back corner of the room. I feel I need to be more closer to the pt.*  *I felt I was quite far away from the scenario* |
| **Module 4** | Scenario was confronting | *It's a bit confronting situation and emotional*  *How sad that a situation like this can be*  *Sometimes the scenario was confronting, but it gave me a better understanding of the reality and how practice should be based* |

**Appendix 1: Qualitative data of intervention students’ responses to open ended questions on the surveys.**
